# Premalignant and malignant lesions in endometrial polyps in patients undergoing hysteroscopic polypectomy

**DOI:** 10.1590/S1679-45082014AO2764

**Published:** 2014

**Authors:** Marco Antonio Lenci, Vanessa Alessandra Lui do Nascimento, Ana Beatriz Grandini, Walid Makin Fahmy, Daniella de Batista Depes, Fausto Farah Baracat, Reginaldo Guedes Coelho Lopes

**Affiliations:** 1 Hospital Israelita Albert Einstein, São Paulo, SP, Brazil; 2 Universidade Cidade de São Paulo, São Paulo, SP, Brazil; 3 Hospital do Servidor Público Estadual “Francisco Morato de Oliveira”, São Paulo, SP, Brazil

**Keywords:** Histeroscopy, Endometrial neoplasms, Polyps/surgery

## Abstract

**Objective:**

To evaluate the incidence of premalignant lesions and cancer in endometrial polyps, in patients undergoing hysteroscopic polypectomy.

**Methods:**

The results of 1,020 pathological examinations of patients submitted to hysteroscopic polypectomy were analyzed, as well as their diagnostic and surgical hysteroscopy findings. As to their menstrual status, 295 (28.9%) patients were in menacme. Of the total, 193 (65.4%) presented abnormal uterine bleeding, and 102 (34.6%) were asymptomatic with altered endometrial echo on transvaginal ultrasound. Out of 725 (71.1%) postmenopausal patients, 171 (23.6%) were symptomatic (abnormal uterine bleeding), and 554 (76.4%) were asymptomatic with endometrial echo >5.0mm.

**Results:**

Twenty-one (2.0%) patients presented premalignant lesions in the polyps, 13 had simple glandular hyperplasia, of which 5 had no atypia, and eight presented atypia. Eight polyps presented focal area of complex hyperplasia: 4 with atypia and 4 without lesions. Cancer was diagnosed in 5 (0.5%) polyps. Of the 21 polyps that harbored premalignant lesions, 12 were interpreted as benign in diagnostic and surgical hysteroscopy. Of the polyps with cancer, 4 were also histeroscopically interpreted as normal.

**Conclusion:**

Symptomatic polyps in menacme and in all postmenopausal women should be resected and submitted to histopathological examination, since they may have a benign aspect, even when harboring areas of cellular atypia or cancer.

## RESUMO

**Objetivo:**

Avaliar a incidência de lesões precursoras e câncer em pólipos do endométrio de pacientes submetidas à polipectomia histeroscópica.

**Métodos:**

Análise dos resultados de 1.020 exames anatomopatológicos de pacientes submetidas, em hospital público, à polipectomia histeroscópica, e achados em suas histeroscopias diagnóstica e cirúrgica. Em termos de estado menstrual, 295 (28,9%) pacientes encontravam-se na menacme. Do total, 193 (65,4%) apresentavam sangramento uterino anormal e 102 (34,6%) eram assintomáticas, com alteração da medida do eco endometrial à ultrassonografia transvaginal. Das 725 (71,1%) pacientes na pósmenopausa, 171 (23,6%) eram sintomáticas (sangramento uterino anormal) e 554 (76,4%) assintomáticas, com eco endometrial >5,0mm.

**Resultados:**

Vinte e uma (2,0%) pacientes apresentaram lesões precursoras nos pólipos, 13 com hiperplasia glandular simples, das quais 5 sem atipias e 8 com atipias. Oito pólipos apresentavam área focal de hiperplasia complexa, quatro com atipias e quatro sem lesões. Câncer foi diagnosticado em cinco (0,5%) pólipos. Dos 21 pólipos que abrigavam lesões precursoras, 12 foram interpretados como benignos nas histeroscopias diagnóstica e cirúrgica. Quatro dos pólipos com câncer também foram interpretados histeroscopicamente como normais.

**Conclusão:**

Os pólipos sintomáticos na menacme, bem como todos na pós-menopausa, devem ser ressecados para realização de exame anatomopatológico, por poderem apresentar aspecto benigno, mesmo quando abrigam áreas de atipia celular ou câncer.

## INTRODUCTION

Endometrial polyps are localized, pedunculated or sessile tissue growths consisting of a variable amount of glands, stroma and blood vessels.^(1)^ They have a soft consistency, similar to the endometrium, and their surface is dark and shiny. Sometimes they ulcerate, bleed or twist, which may lead to partial or complete necrosis.^(2)^

They originate from anywhere in the uterine cavity, but most of them are attached to the uterine fundus, generally in the cornual area.^(3)^ The prevalence of endometrial polyps in the general population ranges from 6 to 38%, and rarely they are present before menarche. They are more frequent between 40 and 50 years of age, with a gradual increase before this age and a decrease thereafter.^(2)^

Polyps may occur in places where there is an increased expression of estrogen receptors, reduced expression of progesterone receptors, or both.^(4,5)^ The concentration of both estrogen and progesterone receptors in the endometrial polyp is higher in the glandular epithelium than in the stroma, as well as in normal endometrium.^(6)^

Although infrequent, polyps can become malignant. To be considered a primary site of malignancy, the tumor must be confined to the apex, with no lesion in its base, as well as in the surrounding endometrium.^(7)^

The prevalence of premalignant lesions and cancer in endometrial polyps is low, but there are studies that try to relate various clinical and epidemiological parameters to the occurrence of malignancy associated with polyps, such as: age, years after menopause, obesity, arterial hypertension, *diabetes mellitus*, hormone therapy, use of tamoxifen, size of polyps, and abnormal uterine bleeding.^(8–11)^ However, there are those who advocate the idea that this increased risk occurs only when these data are considered together and not separately.^(12)^ Endometrial polyps should also be pointed out as a risk factor for the presence of endometrial adenocarcinoma, which is nine times more frequent in patients with polyps than in patients with no polyps.^(13)^

There are several resources for the diagnosis of endometrial polyps, and transvaginal ultrasonography, with or without Doppler flowmetry, is considered the gold standard among noninvasive diagnostic methods. Among the methods considered invasive, outpatient hysteroscopy is the best method for diagnosing endometrial polyps, with 95.6% agreement between the image and the pathological diagnosis.^(14)^ Using hysteroscopy, it is possible to quantify the polyps, analyze the vascularization of their surface, their shape, location, size and width of the implantation base, and it also makes feasible a directed or guided biopsy.^(2,9,15)^

As to management, some authors advocate the systematic removal of all symptomatic polyps.^(2,5,7,13,15–18)^ Therefore, the best option is an operative hysteroscopy, for it is a quick procedure with few risks, presenting excellent cost-effectiveness.^(19)^

## OBJECTIVE

To evaluate the incidence of premalignant lesions and cancer in endometrial polyps in patients undergoing hysteroscopic polypectomy.

## METHODS

The study was approved by the Research Ethics Committee of the *Hospital do Servidor Público Estadual “Francisco Morato de Oliveira”* and registered under number 037/08.

In a retrospective analysis, the medical records of 1,020 consecutive patients who underwent hysteroscopic polypectomy, performed by the Division of Gynecologic Endoscopy, Service of Gynecology, Department of Gynecology and Obstetrics of the *Hospital do Servidor Público Estadual “Francisco Morato de Oliveira”*, in the period of August 1988 to January 2010.

The outpatient operative hysteroscopy reports were analyzed and compared to the results of pathological examinations, which confirmed they were all endometrial polyps. The suspected cases that were not confirmed by histopathological examination were excluded. Polyps with simple or complex hyperplasia without atypia, or with mild atypia, were considered as polyps with premalignant cancer lesions; and polyps with complex hyperplasia and severe atypia were considered as polyps with cancer. Moreover, the interpretation results of polyps were analyzed and specifically compared to the premalignant and malignant lesions.

Postmenopausal status was defined as the absence of menstruation for a period ≥1 year, excluding any conditions or use of medications that could determine this fact.

All surgeries were performed under epidural or spinal anesthesia, using a 9.0mm caliber resectoscope with a handle at the distal extremity, connected to equipment with high-frequency monopolar electrical current (90 Watts). Uterine distension was performed with a 1.5% glycine solution or a 3.0% mannitol solution, controlling the infusion with an electronic hysteroscopy pressure pump, and the intracavitary pressure was maintained between 100 and 150mmHg. A videocamera system was used and allowed recording surgeries on videotape and, more recently, on DVD.

## RESULTS

To study the risk factors for the presence of polyps, the following parameters were analyzed and compared, separately and together: age, menstrual status (whether the patient was in menacme or postmenopause), age at menopause, presence of bleeding after menopause, time after menopause, and endometrial thickness measurement.

The patients' mean age and standard deviation (SD) was 56.5±10.4 years, with a minimum of 24 and a maximum of 88 years, with a 95%confidence interval (CI) between 55.9 and 57.2 years. The age distribution curve was normal, confirmed by the Kolmogorov-Smirnov test, with maximum deviation of 0.0383 and p<0.1.

A total of 1,020 patients were classified into three groups according to the results of the pathological examinations of the polyps, as follows: normal (N) in 994 patients (97.5%); premalignant lesion (P) in 21 patients (2.0%); and cancer (C) in 5 patients (0.5%).

The mean age of each of the three groups was: 56.5±10.4 years (95% CI: 55.8 to 57.1) for N; 56.7±12.9 (95% CI: 50.8 to 62.6) for P; and 63.6±6.5 (95% CI: 55.5 to 71.7) for C. Although group C had greater mean age than groups N and P, there were no statistical differences among the three groups (F=1.17, p=0.3096).

Among 1,020 patients, 295 (28.9%) were in menacme, mean age 45.2 years (range 24-60 years); and 725 (71.1%) were postmenopausal women, mean age 60.9 years (range 41-88 years). The age at menopause ranged from 35 to 64 years, mean 49.4 years. A total of 145 (14.2%) patients had late menopause (above 52 years). Considering the three groups, 707 patients were in group N, 13 in P, and 5 in C. The age at menopause values were: 49.4±4.2 (95% CI: 49.0 to 49.7) for N; 50.4±2.6 (95% CI: 48.8 to 51.9) for P; 52.0±3.5 (95% CI: 47.7 to 56.3) for C. There was a rise in the mean age at menopause in the group P, and the values were even higher in group C, but with no statistically significant differences among the means of the three groups (F=1.33, p=0.2642).

As to the symptoms among the 1,020 patients studied, 364 (35.7%) had abnormal uterine bleeding, of which 193 (53.0%) were in menacme, and 171 (47.0%) were postmenopausal. Of 656 (64.3%) asymptomatic patients with abnormal endometrial thickness measurement (> 5mm) on transvaginal ultrasonography, 554 (84.5%) were in postmenopause, and 102 (15.5%) were in menacme. Among the patients of the *Hospital do Servidor Público Estadual “Francisco Morato de Oliveira”* who were postmenopausal, an endometrial thickness up to 5mm was considered normal.^(20)^ Among the 295 patients of reproductive age, it was observed that 193 (65.4%) complained of abnormal uterine bleeding, and 102 (34.6%) were asymptomatic. Of the 725 postmenopausal patients, 554 (76.4%) were asymptomatic, and 171 (23.6%) had genital bleeding. Considering now the three groups, postmenopausal bleeding occurred in 162 (16.2%) of 994 patients in group N, in 7 (33.3%) of 21 patients in group P, and in 2 (40.0%) of 5 patients in group C.

As to the transvaginal sonographic findings, 629 (61.6%) patients had endometrial thickening; 170 (16.7%) had abnormalities suggestive of polyps; 112 (11.0%) had a suspected submucosal myoma; in 53 (5.2%), the examination was considered normal; in 56 (5.5%), there was no reference to the endometrium. The endometrial echo measurement, which ranged from 2.0 to 34.0mm, was performed in only 889 of the 1,020 patients – 863 in group N; 21 in group P; and 5 in group C. The overall mean thickness and respective SD for the 889 patients was 10.7±4.9mm (95% CI: 10.4 to 11.0). The values of endometrial thickness for each group were: 10.7±4.9mm (95% CI: 10.4 to 11.0), equal to the overall mean, for group N; 11.9±5.3 (95% CI: 9.5 to 14.3) for group P; and 12.8±4.4 (95% CI: 7.3 to 18.3) for group C. There was an increasing arithmetical mean value of thickness as we moved from the group N to the group C, although there was no statistically significant difference among the three groups (F=1.09, p=0.3376).

Table 1 shows the results of the pathological examinations, obtained from the material resected.

**Table 1:** Pathological examinations results

Anatomopathologic finding	n (%)
Endometrial polyp	994 (97.5)
Polyp with simple or complex glandular hyperplasia, with no atypia or with mild atypia	21 (2)
Polyps with areas of complex hyperplasia with severe atypia	1 (0.1)
Well differentiated endometrioid endometrial adenocarcinoma grade I originating from the endometrial polyp	4 (0.4)
Total	1,020 (100)

The findings observed on the outpatient hysteroscopy are shown in table 2.

**Table 2:** Outpatient hysteroscopy results

Outpatient hysteroscopy	n (%)
Endometrial polyp	980 (96.1)
Submucosal myoma	28 (2.7)
Simple endometrial hyperplasia	7 (0.7)
Suspected malignant polyp lesion	5 (0.5)
Total	1,020 (100)

Compiling all polyps, premalignant lesions, and cancer occurrences, there were 26 cases among the 1,020 studied. Using outpatient hysteroscopy performed by different observers, and various resources, over the period of several years of this study, through several stages, 10 (38.5%) of these lesions were diagnosed.

To analyze the correlation between outpatient hysteroscopy and the results of the pathological examinations, we combined polyps, premalignant and malignant lesions into a single category. Thus, 1,020 patients could be dichotomized according to the pathological examination, as follows: 994 women with benign polyps, and 26 women with premalignant and malignant polyps. Similarly, the ambulatory hysteroscopy results were also divided by the same binary or dichotomized criterion: benign polyps, and polyps of premalignant and malignant nature.

The calculation of the agreement was made using a 2x2 table and the application of Bayes' theorem. The calculation results showed a sensitivity of 38.5%, specificity of 96.4%, a positive predictive value of 21.7%, and a negative predictive value of 98.4%. The agreement was 94.9% and the disagreement was 5.1%. The value of Cohen's kappa coefficient was k=0.2535, which was less than the value k=0.4000, thus revealing insufficient agreement.

Table 3 shows the results obtained on operative hysteroscopy.

**Table 3:** Operative hysteroscopy results

Operative hysteroscopy	n (%)
Endometrial polyp	990 (97)
Simple endometrial hyperplasia	17 (1.7)
Submucosal myoma	13 (1.3)
Total	1,020 (100)

In conditions that were similar to outpatient hysteroscopy, we conducted a study with the results of the operative hysteroscopy. This has identified 8 (30.8%) of the 26 lesions found in the pathological examination. By the same methodology, the calculation performed between operative hysteroscopy and pathological examination had the following results: sensitivity 30.8%, specificity 97.7%, positive predictive value 25.8%, and negative predictive value 98.2%. The agreement was 96% and the disagreement 4.0%. The calculation of Cohen's kappa coefficient (k=0.2602) was also considered an insufficient agreement.

All patients with malignancy and complex atypia underwent classical chemotherapy and have had no recurrence of the disease up to the present date.

## DISCUSSION

The advent of transvaginal ultrasonography in daily gynecological practice has brought a significant increase in early diagnosis of endometrial polyps, particularly in asymptomatic patients.^(14)^ In addition, with the use of hysteroscopy, currently considered the gold standard in the evaluation and treatment of diseases of the uterine cavity, it was possible to better identify the risk of endometrial polyps harboring premalignant or malignant lesions.^(14,18)^ Although this potential is variable, their mere presence has revealed a significant increase in risk for the presence of endometrial adenocarcinoma.^(13)^ In this retrospective study of 1,020 patients who underwent hysteroscopic polypectomy, the malignant potential of endometrial polyps was 2.5%. In published studies, the incidence of malignant disease confined to the polyps ranged from 0.5% to 4.8%.^(4,9,13,15,17,21–24)^

Regarding the analysis of the age at menopause, despite an increase in the mean age of the group N to P, and an even higher value in group C, there was no statistically significant difference among the three groups. However, it is worthy mentioning that 3 of the 5 women with cancer in polyps had a late menopause, having had longer exposure to the estrogen stimuli, which corroborates some studies.^(9,10)^

Abnormal uterine bleeding was more frequent in patients of reproductive age, contrary to those who were postmenopausal, most of whom were asymptomatic, and these data are similar to the literature.^(3,6,7,25,26)^ Analyzing the three groups, it was observed that both patients with premalignant lesions (group P) and patients with cancer (group C) were mostly asymptomatic. These results differ from those found by some authors, who advocate the idea that asymptomatic patients have negligible risk of developing malignancy in endometrial polyps.^(15,17,27)^ This difference may be explained by the fact that the patients analyzed in this study have had their polyps diagnosed earlier, when undergoing their routine checkups; therefore, they were in a phase that still presented no bleeding.

As to evaluation of endometrial thickness measurements of 1,020 patients, of 21 with premalignant lesions, and of 5 with cancer in polyps, the respective values were: 10.7mm, 12.2mm, and 13.2mm, *i.e.*, similar to those found in the literature.^(28)^ The analysis of the three groups showed increasing values as we moved from the group N to the groups P and C, although no statistically significant difference was found.

An interesting aspect to be highlighted is the analysis of the interpretations of the polyps by the examiners on outpatient and operative hysteroscopies. In the present study, the sensitivity of outpatient hysteroscopy can be considered low, whereas the specificity was high. Consequently, the overall agreement was good. The Cohen's kappa coefficient revealed insufficient agreement. However, it must be taken in consideration that this type of calculation is hampered by the small proportion of cases in groups P and C, relative to the benign cases. On the other hand, the sensitivity of the operative hysteroscopy was lower than the sensitivity of the diagnostic hysteroscopy. However, considering all values, there was a slightly greater agreement, as well as the value of Cohen's kappa coefficient, in spite of an insufficient agreement.

This inaccuracy in the interpretation depends, besides the experience of the examiner, on the shape of the polyp, the type of vascularization on its surface, and the benign aspect that some of them may display, despite harboring areas of premalignant or malignant tissue. Thus, our findings endorse the assertion that a mere benign hysteroscopic aspect does not ensure the absence of cellular atypia or malignancy in endometrial polyps.^(7)^ Moreover, the differentiation between a polyp with malignant transformation and a polypoid endometrial carcinoma is made by a histological examination of its base.^(29)^

There is no consensus in the literature on the action to be taken in the presence of endometrial polyps. In asymptomatic patients in menacme or postmenopause, and in patients without atypia in the pathological examination of the biopsies obtained on outpatient hysteroscopy, the management may be conservative, if the Doppler flowmetry is normal.^(30)^ The same applies to polyps ≤0.7 cm, which may regress spontaneously.^(31)^

For having found premalignant and malignant lesions in premenopausal and postmenopausal women, with no definitive answer that could explain the appearance of endometrial polyps and in the absence of methods to diagnose a malignant structure exhibiting a benign hysteroscopic aspect, some authors advocate the systematic removal of all polyps at any stage of life.^(15,19,29,32)^

After the development of smaller caliber and more delicate instruments, which made possible the concept of “see and treat”, the management of this condition started to become less controversial. Thus, polyps of up to 2.0cm can be removed at the time of the diagnosis, in an outpatient clinic setting, needing no hospitalization and anesthesia.^(33)^

Based on the results of this study, we believe that all patients of reproductive age with abnormal uterine bleeding, as well as all postmenopausal patients, symptomatic or asymptomatic, should undergo a hysteroscopic polypectomy, endorsing what has been proposed by some authors.^(24,34,35^)

Due to the number of cases and for being retrospective, this study does not make it feasible to establish with certainty which factors could have contributed to the development of premalignant or malignant lesions in endometrial polyps.

Further studies are needed to try to understand the genesis and behavior pattern of polyps, as well as their possible malignant transformation.

## CONCLUSION

Symptomatic polyps in premenopausal and in all postmenopausal patients should be resected and submitted to histopathological examination, as they may appear to be benign, even when harboring areas of cellular atypia or cancer.
